# Therapeutic Efficacy of an Anti-P116-661 Polyclonal Antibody Against *Mycoplasma pneumoniae* Infection

**DOI:** 10.3390/pathogens14101038

**Published:** 2025-10-13

**Authors:** Yiting Zhang, Xinqi Geng, Yan Liu, Wenli Li, Feng Shao, Mengmeng Jin, Jinzhi Wang, Linding Wang

**Affiliations:** 1 Anhui Provincial Key Laboratory of Zoonoses, Department of Microbiology and Parasitology, School of Basic Medical Sciences, Anhui Medical University, Hefei 230032, China; ytzhang181@163.com (Y.Z.); gengxinqi2023@163.com (X.G.); 15727016005@163.com (Y.L.); lwl20201125@163.com (W.L.); 2 Department of The Second Clinical School of Medicine, Anhui Medical University, Hefei 230032, China; 19855653336@163.com (F.S.); 19011307291@163.com (M.J.); 3 Department of Basic Medical Sciences, Anhui Medical University, Hefei 230032, China; w20030415@126.com

**Keywords:** *Mycoplasma pneumoniae* (MP), P116-661 polyclonal antibody, HE staining, cell adhesion, IL-6, TNF-α

## Abstract

The aim of this study was to investigate the therapeutic potential of a polyclonal antibody against the *Mycoplasma pneumoniae* (MP) P116-661 protein. A polyclonal antibody against the P116-661 protein was obtained by immunizing New Zealand white rabbits, and its therapeutic effects were systematically evaluated by various experimental methods. An immunofluorescence assay was used to detect the inhibitory effect of the P116-661 polyclonal antibody on the adhesion of MP cells to A549 cells. ELISAs and Western blotting were used to analyze the expression levels of inflammatory factors, such as IL-6 and TNF-α, in Beas-2b cells and model mice after MP infection. HE staining was used to observe pathological changes in the lung tissue of the infected mice. The results showed that the P116-661 polyclonal antibody effectively inhibited the adhesion of MP cells to A549 cells. It significantly reduced the secretion levels of inflammatory factors, such as IL-6 and TNF-α, in Beas-2b cells and mice after MP infection. Moreover, the antibody significantly improved the pathological damage to the lungs that was caused by MP infection in mice. This study confirms that the P116-661 polyclonal antibody has good therapeutic effects in vitro and in vivo, providing a new experimental basis for immunotherapy against MP infection.

## 1. Introduction

MP is a pathogen that lacks a cell wall, relies on cholesterol for growth, and is highly polymorphic [1]. Its genome is one of the smallest genomes known in self-replicating organisms, making MP one of the simplest prokaryotes [2,3]. MP can infect people of all ages, causing upper and lower respiratory tract infections and accounting for up to 40% of community-acquired pneumonia cases [4]. In addition to typical respiratory symptoms, MP infection can cause a variety of extrapulmonary clinical manifestations, which involve the skin, central nervous system, kidney, musculoskeletal system, digestive system, and blood system [5,6]. In addition, studies have shown that acute MP infection may be closely related to the occurrence and development of chronic asthma [7].

The pathogenic mechanism of MP involves adhesion and damage to host cells, and it may further invade host cells during infection [8]. The key adhesion proteins include P1, P30, P116, and HMW1-3 [9,10,11]. Currently, the P1 protein is the most extensively studied, whereas the P116 protein is also critical for the pathogenesis of MP due to its unique biological functions, mediating host cell adhesion and facilitating the uptake of cholesterol—a process that is vital for MP cell viability.

In addition, the P116 protein has strong immunogenicity, and a high titer of a polyclonal antibody in the serum can be obtained by immunizing New Zealand white rabbits with the P116 protein as an antigen [12,13]. On the basis of the important function and immunological characteristics of the P116 protein, we chose P116-661 as the research object to explore the potential therapeutic value of its polyclonal antibody.

Since the 1960s, the research and development of MP vaccines have represented a tortuous process. In early studies of inactivated MP vaccines, compared with the unvaccinated control group, some vaccinated patients presented aggravated clinical symptoms during subsequent natural infection, which was referred to as vaccine-enhanced disease (VED) and directly hindered the development of MP vaccines [14]. Therefore, researchers have changed vaccine development strategies to live attenuated vaccines, subunit vaccines (targeting key proteins, such as P1, P30, P116 and CARDS toxins) and DNA vaccines [1]. However, to date, there is no *M. pneumoniae* vaccine licensed for human use worldwide. Considering the lack of breakthroughs in prevention methods, the treatment strategy for MP infection is particularly important. At present, azithromycin, a macrolide antibiotic used as a first-line treatment, can effectively improve the lung function of patients, but, with long-term clinical use, the problem of MP drug resistance has become increasingly prominent [15]. To address this challenge, combination regimens, such as vitamin C and montelukast sodium, are often used in clinical practice [16,17,18].

Although monoclonal antibody technology has seen revolutionary progress in the field of antibody therapy because of its advantages of high specificity, homogeneity, and large-scale production, polyclonal antibodies still have irreplaceable importance in basic research and clinical applications. Compared with monoclonal antibodies, polyclonal antibodies have a shorter development cycle, simpler technical operations, and lower equipment investment [19,20].

More importantly, owing to their multiepitope recognition properties, polyclonal antibodies have shown excellent applicability in important experimental techniques, such as immunoprecipitation, Western blotting, and enzyme-linked immunosorbent assays, which makes them the reagents of choice in many research fields.

In this study, a polyclonal antibody against a *Mycoplasma pneumoniae* P116-661 protein fragment was prepared and found to effectively block the adhesion of *Mycoplasma pneumoniae* cells to respiratory epithelial cells and significantly inhibit the excessive secretion of inflammatory factors that is induced by infection. The results showed that the polyclonal antibody not only effectively reduced the adhesion rate of MP cells to A549 cells but also significantly inhibited the expression of the key inflammatory factors IL-6 and TNF-α in Beas-2b cells and in model mice after infection. These findings not only confirm the potential application of polyclonal antibodies in immunotherapy for MP infection but also provide an important experimental basis for the development of novel anti-infection strategies.

## 2. Materials and Methods

### 2.1. Experimental Materials

The *Mycoplasma pneumoniae* (MP) standard strain (ATCC 15531) was deposited in our laboratory. The recombinant expression plasmid P116-661 and its corresponding polyclonal antibody were independently prepared in our laboratory. A human bronchial epithelial cell line (Beas-2b) and a human lung cancer epithelial cell line (A549), which were routinely cultured in our laboratory, were used in the cell experiments. The enzyme-linked immunosorbent assay (ELISA) kits used were as follows: a human/mouse IL-6 detection kit, a human/mouse TNF-α detection kit, and a human *Mycoplasma pneumoniae* IgG detection kit (Enzyme Immunoassay Industry Co., Ltd., Jiangsu, China). The other reagents were as follows: CCK-8 (Butterfly Biotechnology Co., Ltd., Hefei, China); an immunostaining blocking solution, a DAPI staining solution, and a TMB chromogenic solution (Beyotime Biotechnology, Shanghai, China); an anti-beta-actin antibody (Servicebio Biotechnology Co., Ltd., Wuhan, China); a TNF-α rabbit pAb (ABclonal Bio, Hefei, China); and an FITC-labeled donkey anti-rabbit IgG and HRP-labeled goat anti-rabbit IgG (Sangon Biotech, Ltd., Shanghai, China). Experimental New Zealand white rabbits and BALB/c mice were provided by the animal center of our laboratory.

### 2.2. Preparation of P116 Protein and Its Polyclonal Antibody

#### 2.2.1. Preparation of Polyclonal Antibody Against P116-661

The recombinant plasmid pQE80L-P116-661 was constructed and preserved in our laboratory (Information on the pQE80L-P116-661 recombinant plasmid can be found in the Supplementary Material). Protein expression was induced with 1 mM isopropyl β-D-1-thiogalactopyranoside (IPTG) for 4 h at 37 °C. Following induction, inclusion bodies were harvested via ultrasonication and centrifugation. The solubilized proteins were subjected to immobilized metal affinity chromatography (IMAC) using a pre-packed Ni-NTA agarose column. After binding, the column was washed with 50 mM imidazole to eliminate non-specifically bound contaminants. Target proteins were subsequently eluted with 250 mM imidazole. The purified P116-661 protein was collected, buffer-exchanged, and stored at −80 °C for subsequent applications.

Four healthy adult New Zealand white rabbits (2.5–3.0 kg) were randomly divided into an experimental group (*n* = 3, P116-661-1 to P116-661-3) and a control group (*n* = 1). The rabbits in the experimental group were immunized with the recombinant protein P116-661, and that in the control group was treated with an equal volume of PBS. The immunization process consisted of three stages. For the first immunization, 300 μg of the purified P116-661 recombinant protein was emulsified with an equal volume of complete Freund’s adjuvant, and then immunization was performed by the subcutaneous injection of P116-661 through multiple points on the back of the rabbit. Two weeks later, 200 μg of the antigen was emulsified with incomplete Freund’s adjuvant and injected in the same way. From the third week, 100 μg of the purified protein was injected into the ear vein every week. Blood samples from the rabbits in the experimental group with confirmed antibody production were collected by cardiac puncture under isoflurane anesthesia. The serum was separated and filtered through a 0.22 μm filter membrane to remove bacteria. The serum of the rabbits from the control group was prepared simultaneously. Immunoreactivity was subsequently analyzed by Western blotting and ELISA [21,22].

#### 2.2.2. Characterization of Polyclonal Antibody by Western Blotting and Titer Determination by ELISA

Following electrophoresis, P116-661 proteins were transferred to PVDF membranes. The membranes were blocked with 5% BSA in TBST for 2 h at room temperature and subsequently incubated overnight at 4 °C with P116-661 polyclonal antibody at a dilution of 1:400 (unimmunized rabbit serum was used in the control group). After three 10 min washes with TBST, the membranes were incubated with goat anti-rabbit IgG (1:5000) for 2 h at room temperature for signal detection.

The P116-661 purified recombinant protein was diluted to 2.5 ng/μL by 0.05 mol/L pH 9.6 carbonate buffer and then added to 96-well plates with 50 μL at 4 °C overnight. The next day, the 96-well plates were washed with PBST 3 times, for 10 min each time. After washing, 200 μL blocking solution was added to each well, and they were incubated at 37 °C for 90 min and then washed again. Serum samples diluted in the blocking solution (range: 1:200–1:25,600) were added to each well at 50 μL and incubated at 37 °C for 1 h. The goat anti-rabbit IgG antibody diluted with blocking solution at 1:5000 was added to the plates at 50 μL per well and incubated at 37 °C for 1 h. The plates were washed three times, for 10 min each time. TMB was added to the plates at 50 μL per well. After 5 min of reaction at 37 °C, a stop solution was added at 50 μL per well. The plates were read at 405 nm on an automated ELISA plate reader. All data were collected and 3 replicate trials were conducted on all controls and samples.

### 2.3. Inhibition of MP Adhesion Assay with the P116-661 Polyclonal Antibody

The inhibitory effect of the P116-661 polyclonal antibody on the adhesion of MP cells to A549 cells was evaluated by an immunofluorescence assay. The experimental procedure was as follows: the MP bacterial solution was incubated with different dilutions of P116-661 polyclonal antibody (1:100, 1:200, 1:400, and 1:800) for 12 h. At the end of the incubation, the host cells were washed three times with PBS and fixed with 4% paraformaldehyde for 10 min, followed by washing again. The cells were then permeabilized with 0.3% Triton X-100 for 10 min, washed with PBS, and blocked with an immunofluorescence blocking solution for 1 h at 37 °C.

The treated cell slides were transferred to plates, and 50 μL of the P116-661 polyclonal antibody was added. Each concentration was set up in three wells. Moreover, the azithromycin group, the azithromycin combined with vitamin C group, and the azithromycin combined with montelukast sodium group were used as positive controls. After overnight incubation in a wet box at 4 °C, the cells were washed three times with PBS and subsequently incubated with 50 μL of FITC-labeled donkey anti-rabbit IgG (diluted 1:100), and the slides were incubated for 1 h at 37 °C in the dark, followed by washing. DAPI containing a quenching agent was finally added for 5 min, after which the samples were washed with PBS and observed under the THUNDER Imager 3D assay (Leica) or confocal microscopy (Zeiss).

The resulting fluorescence intensity was positively correlated with the amount of MP adhesion. The lower the dilution of the polyclonal antibody (the higher the concentration of the antibody), the weaker the green fluorescence, indicating a more significant inhibitory effect of the antibody on MP adhesion. The experimental data were quantitatively analyzed in terms of fluorescence intensity using the ImageJ fiji 2.14.0 software and statistically analyzed using the graghpaid 8.0.

### 2.4. Beas-2b Cell Infection and Treatment

#### 2.4.1. MP Infection Experiment

Beas-2b cells were used to establish an MP infection model. The cells were seeded in 6-well plates and cultured for 12 h. MP cells were adjusted to three infection doses, namely 5, 25, and 100 MOI. One hundred microliters of the MP bacterial suspension with different MOIs was added, and the mixtures were cultured for 12 h at 37 °C. Uninfected cells were used as a negative control. At the end of incubation, the Beas-2b cells were washed three times with PBS, and proteins were extracted using lysis buffer for Western blot analysis.

#### 2.4.2. Screening for Optimal Drug Concentrations

The effects of the drugs on cell viability were determined by a CCK-8 assay. The experimental procedure was as follows. After grinding azithromycin, vitamin C, and montelukast sodium tablets, stock solutions were prepared with sterile PBS and filtered through a 0.22 μm filter membrane for sterilization. A series of concentration gradients were prepared using twofold serial dilution. Azithromycin and vitamin C were diluted to five concentrations (100, 50, 25, 12.5, and 6.25 μg/mL), and the concentrations of montelukast sodium were 10, 5, 2.5, 1.25, and 0.625 μg/mL.

Beas-2b cells were seeded in 96-well plates, and, after 12 h of culture, 10 μL of the different concentrations of the drug solutions was added to each well, with each concentration added to 3 wells. Moreover, untreated cells were used as controls. After 12 h of incubation at 37 °C, 10 μL of the CCK-8 reagent was added to each well, and incubation continued for 20 min. Cell viability was calculated by measuring the OD450 nm values with a microplate reader.

#### 2.4.3. Determination of Optimal Concentration of Each Drug Mixture

After the optimal concentration of each drug was determined, combinations of vitamin C and montelukast sodium with azithromycin were added to 96-well plates, and cell viability was measured to ensure that it was not decreased by the drug combinations.

#### 2.4.4. Infection of Beas-2b Cells and Drug Addition

The bacterial suspension of MP was added to the 96-well plates and incubated with the cells for 12 h at 37 °C. At the end of the incubation, the medium was discarded, followed by the addition of fresh medium and 10 μL of the polyclonal antibody. Then, solutions of azithromycin, azithromycin combined with vitamin C, and azithromycin combined with montelukast sodium were added to each well, followed by incubation at 37 °C for 6 h. The remaining steps were the same as above.

Proteins were subsequently isolated and subjected to Western blotting.

### 2.5. Infection and Treatment of BALB/c Mice

#### 2.5.1. Mouse Model of MP Infection

Thirty 8-week-old SPF male BALB/c mice (weighing approximately 30 ± 5 g) were randomly divided into an experimental group and a control group. The experimental group was divided into 10^5^ CCU/mL and 10^3^ CCU/mL groups, with 10 mice in each group. After grouping, the mice were inoculated intranasally with MP. First, the mice were anesthetized with isoflurane isoflurane, and then 20 μL of the MP bacterial suspension at concentrations of 10^5^ and 10^3^ CCU/mL was slowly dropped into the nasal cavity of each mouse. The mice in the control group were treated with MP medium once a day for 3 days. Subsequently, the mice were anesthetized with isoflurane, and the tracheae were exposed with scissors. Normal saline was slowly injected into the trachea, after which the lung fluid was slowly withdrawn to obtain alveolar lavage fluid. The presence of MP in the bronchoalveolar lavage fluid was detected by PCR immediately after collection to confirm the successful establishment of the model. Primer information: P116-661-r:5′-AACTGGATCCATGAGTGCTATTATCTCCCTATCAGTCGCT-3′; P116-661-f:5′-AGGTCGACTTACTACTTGATCAGCCATAAA-3′. Target gene GenBank accession no. CP179138.1.

#### 2.5.2. Treatment of MP-Infected Mice with the Polyclonal Antibody and Each Drug

For modeling, the mice were divided into a control group and an experimental group. In accordance with the results of the above pre-experiment with drug dilutions, the experimental group ultimately received a 25 μg/mL azithromycin solution (700 μL), 25 μg/mL vitamin C solution (550 μL), or 10 μg/mL montelukast sodium solution combined with a 50 μg/mL azithromycin solution (460 μL) via tail vein injection, with a 1:100 dilution (500 μL) of the polyclonal antibody injected into the tail vein of each mouse, and sterile PBS (500 μL) was used as the control. Eyeball blood was collected for preservation after 3 consecutive days of injection. The left and right lung lobes of the mice were collected at the same time to be used for Western blotting and HE staining, respectively.

#### 2.5.3. Protein Extraction from the Lung Tissue of the Left Lobe

The lung tissue of the left lobe of each mouse was cut into small pieces and placed into magnetic bead tubes, and a precooled lysate (Tiangen, Beijing, China) containing an inhibitor was added. This mixture was homogenized with a homogenizer until the tissue was completely lysed. The lysed samples were centrifuged at 12,000 rpm (ThermoFisher, am Kalkberg, Germany) for 15 min at 4 °C. A total of 100 μL of the supernatant was aspirated, protein loading buffer was added, and the samples were boiled for 10 min for Western blot analysis.

#### 2.5.4. HE Staining of the Lung Tissue of the Right Lobe

The right lobe of the lung of each mouse was fixed with 4% paraformaldehyde for more than 24 h, and the fixed tissue was placed in a dehydration box in a dehydrator and dehydrated in gradient alcohol. The tissue soaked in wax was placed in an embedding machine and cooled on a freezing table at −20 °C. The cooled wax blocks were subsequently sliced on a paraffin slicer and baked in an oven at 60 °C. The sections were deparaffinized and stained with hematoxylin (Solarbio, Beijing, China) for 5 to 10 min to render the nuclei blue; they were then counterstained with eosin (Solarbio, Beijing, China) for 10 to 30 s to render the cytoplasm pink. Finally, the cells were dehydrated through an ethanol gradient (50%, 70%, 80%, 95%, and 100%), cleared in xylene, and mounted with a cover slip, after which the slices were sealed with neutral gum to observe the results.

### 2.6. Statistical Analysis

All statistical analyses were performed with the GraphPad Prism 8 software.

## 3. Results

### 3.1. Titer Detection and Immunoreactivity Analysis of Polyclonal Antibodies

**Figure 1:**
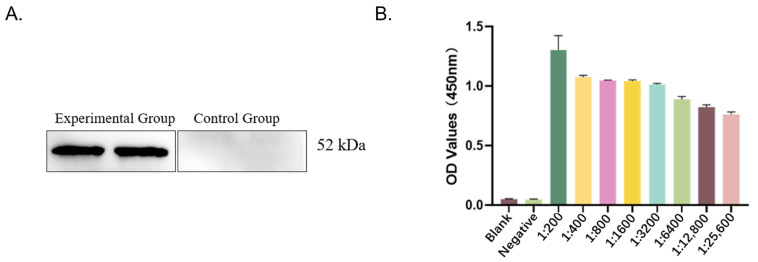
Detection of immune reactivity and titer. (**A**) Results of immune reactivity analysis (experimental group: immunized rabbit serum; control group: unimmunized rabbit serum). (**B**) Results of titer detection.

To evaluate the specificity of the P116-661 polyclonal antibody, serum from unimmunized rabbits was used as the primary antibody in the control group, while serum from immunized rabbits was used in the experimental group. The results demonstrated that the serum from the experimental group specifically recognized the target antigen, whereas no reactivity was observed with the control serum (Figure 1A).

The specificity of the P116-661 polyclonal antibody was validated, and its titer was determined. The serum was considered positive when the OD_450_ value exceeded 1.0. The titer was defined as the highest serum dilution that yielded a positive result. As summarized in Table 1 and illustrated in Figure 1B, the polyclonal antibody titer was determined to be 1:3200.

### 3.2. MP Induces Expression and Secretion of IL-6 and TNF-α in Bronchial Epithelial Cells

**Figure 2:**
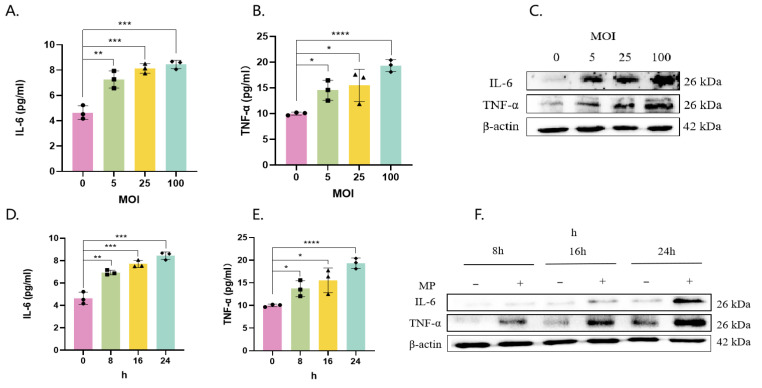
MP induces IL-6 and TNF-α expression and secretion in bronchial epithelial cells. Beas-2b cells were infected with MP at MOIs of 0, 5, 25, and 100 for 16 h (**A**–**C**) or at an MOI of 100 for 8–24 h (**D**–**F**). Cell supernatant secretion levels and protein expression levels were determined by ELISA (**A**,**B**,**D**,**E**) and Western blotting (**C**,**F**). The results of 3 independent experiments were analyzed statistically by *t*-test, *: *p* < 0.05, **: *p* ≤ 0.01, ***: *p* ≤ 0.001, ****: *p* ≤ 0.0001, ns: *p* ≥ 0.05, Squares, diamonds, rectangles and triangles represent different compound holes in the same group. The same is true in the subsequent figure.

The levels of IL-6 and TNF-α in the supernatant (Figure 2A,B) and those of cellular proteins (Figure 2C,F) in Beas-2b cells infected with MP increased in a manner dependent on both the multiplicity of infection (MOI) (Figure 2A–C) and time (Figure 2D–F).

### 3.3. Determination of the Optimal Concentration of Each Therapeutic Drug

**Figure 3:**
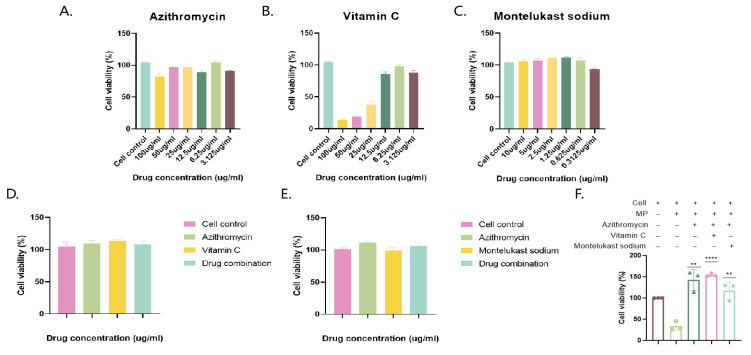
Determination of optimal drug concentrations and MP infection and drug therapy. Azithromycin, vitamin C, and montelukast sodium were added to cells after gradient dilution to determine cell viability (**A**–**C**), and then azithromycin and vitamin C were added to cells in combination, and azithromycin and montelukast sodium were added to cells to determine cell viability (**D**,**E**). Cell viability after treatment with MP bacteria and drugs (+, with drug; −, without drug). (**F**) The results of 3 independent experiments were expressed and analyzed statistically by *t*-test.

Beas-2b cells were incubated with different concentrations of azithromycin and montelukast. The optimal drug concentrations that did not affect cell viability were 6.25 and 1.25 μg/mL, respectively, and the combination of the drugs did not affect cell viability (Figure 3).

### 3.4. Detection of Cytokine Levels After Polyclonal Antibody and Drug Addition

**Figure 4:**
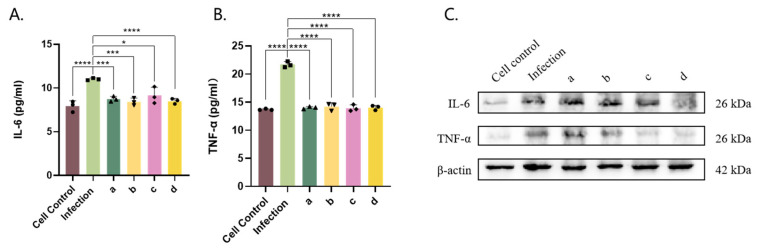
Determining the optimal concentrations of each drug and MP infection and drug treatment. Azithromycin treatment group (a), azithromycin-assisted vitamin C treatment group (b), azithromycin-assisted montelukast sodium treatment group (c), and anti-P116-661 polyclonal antibody group (d) were incubated with MP added to the Beas-2b cell culture medium to detect the content of each cell factor after MP infection (**A**–**C**). The results of 3 independent experiments were expressed and analyzed statistically by *t*-test.

The results showed that the polyclonal antibody had a significant effect in terms of reducing IL-6 and TNF-α secretion (Figure 4A,B) and expression (Figure 4C).

### 3.5. Inhibition of MP Adhesion to A549 Cells by the P116-661 Polyclonal Antibody

**Figure 5:**
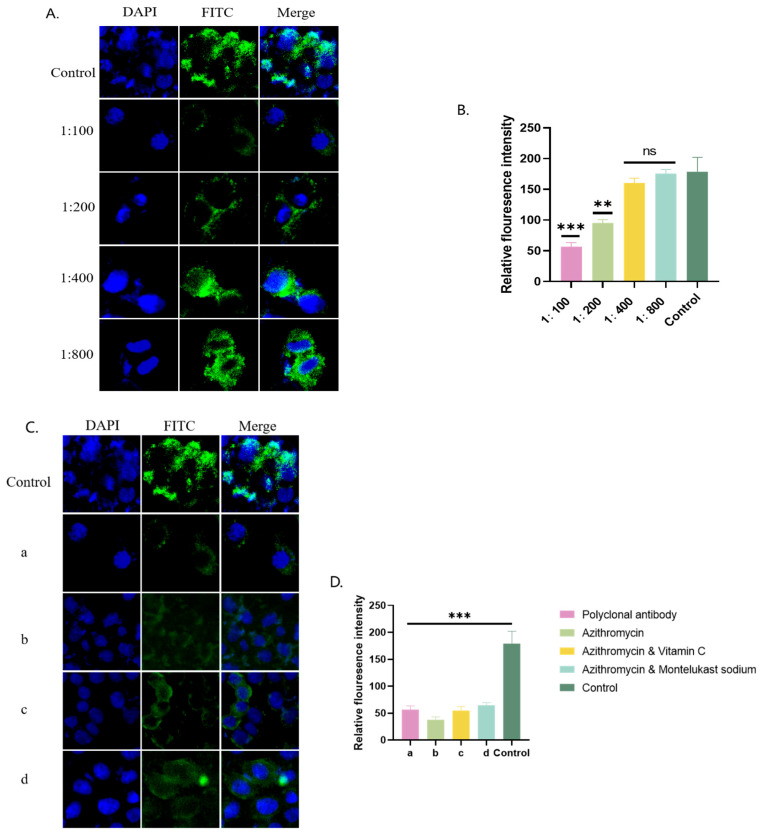
P116-661 adhesion inhibition experiment. (**A**) Immunofluorescence microscopy results for polyclonal antibodies at different dilutions. (**B**) Immunofluorescence intensity values corresponding to A. (**C**) Immunofluorescence microscopy results of polyclonal antibody group (a), azithromycin group (b), azithromycin combined with vitamin C group (c), and azithromycin combined with montelukast sodium group (d). (**D**) Immunofluorescence intensity values corresponding to C. The results of 3 independent experiments were expressed and analyzed statistically by *t*-test.

Green fluorescence was observed under a fluorescence microscope when the P116-661 polyclonal antibody was incubated with A549 cells. The lower the dilution of the polyclonal antibody, the lower the amount of green fluorescence and the lower the fluorescence intensity value, which indicated the inhibition of the adhesion of MP cells to A549 cells. The results showed that the P116-661 polyclonal antibody could significantly inhibit the adhesion of MP cells to A549 cells (Figure 5).

### 3.6. Construction of the Mouse Model of MP Infection

**Figure 6:**
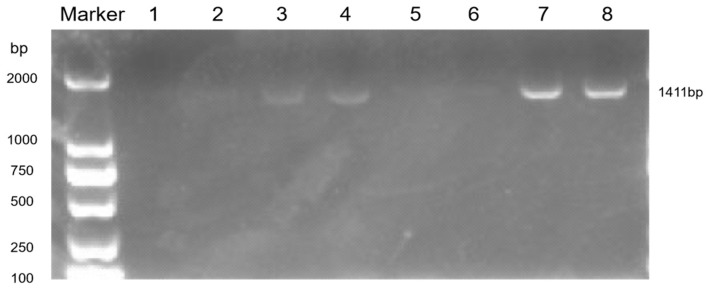
PCR detection of lung tissue homogenate.

The bronchoalveolar lavage fluid and tissue homogenates of the mice were collected for the PCR detection of MP (Figure 6). There were obvious bands at 1411 bp in lanes 3, 4, 7, and 8, indicating that the mouse model of *Mycoplasma pneumoniae* infection was successfully established.

The values were 1:10^5^ CCU/mL alveolar lavage fluid in the control group, 2:10^3^ CCU/mL alveolar lavage fluid in the control group, 3:10^5^ CCU/mL alveolar lavage fluid in the infection group, 4:10^3^ CCU/mL alveolar lavage fluid in the infection group, 5:10^5^ CCU/mL tissue homogenate in the control group, 6:10^3^ CCU/mL in the control group, 7:10^5^ CCU/mL in the infection group, and 8:10^3^ CCU/mL in the infection group.

### 3.7. HE Staining of Lung Tissue and Determination of the Levels of Inflammatory Factors IL-6 and TNF-α in the Supernatant of Eyeball Blood

**Figure 7:**
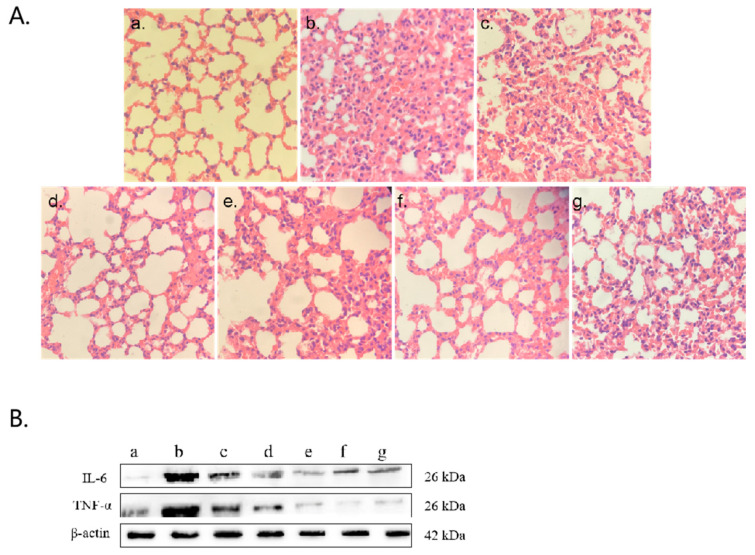

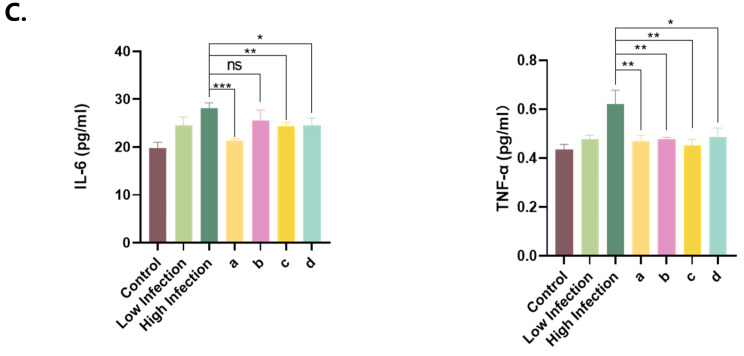
HE staining of lung tissue and detection of inflammatory factors. (**A**) HE staining of lung tissue. (**B**) Western blot detection: (a) control group, (b) 10^3^ CCU/mL infection group, (c) 10^5^ CCU/mL infection group, (d) azithromycin injection group, (e) azithromycin combined with vitamin C injection group, (f) azithromycin combined with montelukast sodium injection group, (g) polyclonal antibody injection group. (**C**) Levels of IL-6 and TNF-α in ocular blood supernatant: (a) azithromycin injection group, (b) azithromycin combined with vitamin C injection group, (c) azithromycin combined with montelukast sodium injection group, (d) polyclonal antibody injection group. The results of 3 independent experiments were analyzed by *t*-test.

The control mice presented a normal alveolar structure in their lung tissue (Figure 7(Aa)). In contrast, the high-MP infection group presented notable pathological alterations, such as thickened alveolar walls, bronchial constriction, and extensive neutrophil infiltration (Figure 7(Ab)). Similar but milder pathological changes were observed in the low-infection group (Figure 7(Ac)). Treatment of the high-infection group with various drug regimens (Figure 7(Ad–f)) significantly ameliorated MP-induced lung injury. Additionally, polyclonal antibody administration (Figure 7(Ag)) effectively mitigated inflammatory infiltration and congestion. The Western blot analysis (Figure 7B) and ELISAs (Figure 7C) corroborated the abilities of the polyclonal antibody and drug treatments to modulate aberrant expression levels of IL-6 and TNF-α.

## 4. Discussion

In established research, experimentally induced MP infection in mice is a well-accepted and widely utilized model for screening therapeutic agents and investigating immune mechanisms, with a particular emphasis on humoral immunity [23,24]. In this study, we employed a standard protocol consisting of intranasal inoculation under anesthesia followed by repeated challenges. The model of MP infection established using this method can be used to evaluate drug efficacy [10,25].

Neutrophil infiltration has been widely confirmed as a typical pathological feature of pneumonia induced by MP infection, and its mechanism is closely related to the cascade of inflammatory factors triggered by MP infection [26,27]. IL-6 and TNF-α are important inflammatory markers [28,29]. In this study, P116-661 polyclonal antibody treatment significantly improved the pathological damage to the lungs of MP-infected mice, as evidenced by a significant reduction in the degree of neutrophil infiltration in lung tissue (Figure 4A) and a significant reduction in the expression levels of the key inflammatory factors IL-6 and TNF-α (Figure 4B). Further in vitro experiments confirmed that the antibody effectively inhibited the adhesion of MP cells in the A549 cell model and significantly reduced the production of MP infection-induced inflammatory factors, such as IL-6 and TNF-α, in the Beas-2b cell model. Notably, the P116-661 polyclonal antibody showed good therapeutic effects compared with those of commonly used drugs in clinical practice. These results indicate that the P116-661 polyclonal antibody has dual therapeutic effects. On the one hand, it has a direct anti-infection effect by inhibiting the cell adhesion of MP; on the other hand, it alleviates the tissue damage caused by MP infection by regulating the inflammatory response, which provides a new potential therapeutic strategy for the clinical treatment of MP pneumonia.

MP infection was once considered a benign and self-limited disease that was highly sensitive to macrolide antibiotics. However, clinical observations have revealed that, even with standard antibiotic treatment, some patients still have persistent fever and progressive clinical deterioration and eventually develop severe *Mycoplasma pneumoniae* pneumonia [30]. Notably, the prevalence of macrolide-resistant MP strains has increased significantly in recent years, especially in China, where the incidence has reached 26.9–100%, posing a serious challenge for clinical treatment [31].

At present, tetracyclines and quinolones are widely used in clinical practice as second-line treatment options, but their use in children is limited owing to potential adverse effects, such as bone development toxicity [32,33]. In this context, antibody-based biotherapeutic strategies have attracted much attention because of their high specificity and good safety profiles. With the development of antibody engineering technology, rationally designed polyclonal antibodies have shown broad clinical application prospects [34].

This study confirmed that, although the P116-661 polyclonal antibody was less effective than traditional antibiotics in the treatment of a macrolide-sensitive strain, it had significant therapeutic effects in terms of improving the inflammatory response and reducing tissue damage after MP infection. Specifically, this antibody effectively inhibited the adhesion of MP cells to host cells, reduced the expression levels of key inflammatory factors (IL-6 and TNF-α), and significantly alleviated pathological changes, such as neutrophil infiltration. These findings suggest new intervention strategies for the treatment of *Mycoplasma pneumoniae* pneumonia and have important clinical translational value.

This study has the following limitations: first, the purification of polyclonal antibodies is difficult; second, there is a risk that heterologous polyclonal antibodies may trigger an immune response in humans. Moreover, their complex components make it challenging to accurately predict immunomodulatory function and allergy risk. In addition, between-batch differences also pose a practical barrier to drug quality control. Future studies will focus on optimizing immunization protocols, refining purification strategies, and employing high-throughput sequencing to characterize the antibody composition. The use of humanized animal models will also be implemented to reduce heterologous immunogenicity.

## Figures and Tables

**Table 1:** Determination of P116-661 polyclonal antibody titer.

No.	Blank Control	Negative			Dilutions of Polyclonal Antibody Serum					
			1:200	1:400	1:800	1:1600	1:3200	1:6400	1:12,800	1:25,600
RA1	0.0531	0.0426	1.1648	1.0907	1.0421	1.0283	1.0045	0.8644	0.8338	0.7834
RA2	0.0502	0.0411	1.3791	1.0712	1.0501	1.0428	1.0177	0.9144	0.7957	0.7567
RA3	0.0498	0.0514	1.0612	1.0612	1.0433	1.0505	1.0189	0.8820	0.8316	0.7448

## Data Availability

The data that support the findings of this study are available from the corresponding author upon reasonable request.

## Supplementary Materials

The following supporting information can be downloaded at: https://www.mdpi.com/article/10.3390/pathogens14101038/s1, Information on the pQE80L-P116-661 recombinant plasmid and restriction sites.
