# Supplementary material for: Therapeutic Efficacy of an Anti-P116-661 Polyclonal Antibody Against Mycoplasma pneumoniae Infection
Source: Pathogens. 2025 Oct 13;14(10):1038. doi: 10.3390/pathogens14101038 (PMC12567429; doi:10.3390/pathogens14101038)
Supplement: Supplementary file 1 [file pathogens-14-01038-s001.zip › pathogens-3859809-supplementary.pdf]

Plasmid information

CTCGAGAAATCATAAAAAATTTATTTGCTTTGTGAGCGGATAACAATTATAATAGA
TTCAATTGTGAGCGGATAACAATTTCACACAGAATTCATTAAAGAGGAGAAATTA
ACTATGAGAGGATCGCATCACCATCACCATCACGGATCCaTGAgtgctattatctccctatcag
tcgctggtactgtgggaacaactgcggtggtagtacctacaactataacgcttgtaaataagacccaccaagtagaacatgaatc
agaacaatcggattttcaagatattcgctttggtcttaatagtgttaagttgccaaaagcacagccagctgcggcaactagaattac
cgtggaaaacgggactgataaattagtcaactataagtcctcaccacaacaactctttttagcgaagaacgcgcttaaggataaac
tccaaggtgagtttgataaattcctaagtgatgcgaaggccttcccagcgctaaccgctgatttacaggaatgggttgaccaacag
ctgtttaatccaaaccaaagtttctttgatttaagtgcgcccaggtcaaactttaccctttcatctgacaaaaaggctagtttagacttt
atcttccgctttactaacttcaccgaatccgttcagttgttaaaactaccagaaggtgtatcggttgtagttgactccaaacaaagctt
tgattactatgtcaatgctagtgcccaaaaattattagttctaccgctgtctttaccagattacactttgggtttaaactatatgtttgac
cacattactttaaacggtaaggttgtcaataaatttagttttaatccgttcaaaacgaatttaaacctcgcctttagcaacgtttacaat
ggcgttgatgtgtttgaagcacaaaagaatttagtaggtaagggtaaatacctcaacacccacgtgaaggctgaagacgtaaag
aaggacgttaatgccaacattaaaaaccaatttgacattgccaaaattatcgctgagctaatgggtaaagcccttaaagaatttgg
caatcaacaagaaggtcaaccattatccttcctaaaggtaatggataaagttaaagaagattttgaaaaactgtttaacttagtccg
tcctggattgggtaaatttgttaaggacttaatccaaagtagtagtcaagcagaaaacaagataactgtctacaagttaatctttga
caacaaaaagaccatcttaaacctacttaaagagctttccattccggaattaaactcttctttaggtttagtggacgtcttgtttgatgt
cattactgactctgatggtctctatgaaaggttgcaatctttcaaagacttaatcgttccagcagttaaaacgaatgaaaaaaccgc
ggctttaagtccattaattgaagagttattaacccaaaaggatacctatgtgtttgacttaattcaaaaacacaagggtatcttgact
aacttgttaaagaacttcttagctgatttccaaaaatcaacgccgtttatggctgatcaagtagTAAGTCGACCTGCAG
CCAAGCTTAATTAGCTGAGCTTGGACTCCTGTTGATAGATCCAGTAATGACCTCAG
AACTCCATCTGGATTTGTTCAGAACGCTCGGTTGCCGCCGGGCGTTTTTTATTGGT
GAGAATCCAAGCTAGCTTGGCGAGATTTTCAGGAGCTAAGGAAGCTAAAATGGA
GAAAAAAATCACTGGATATACCACCGTTGATATATCCCAATGGCATCGTAAAGAA
CATTTTGAGGCATTTCAGTCAGTTGCTCAATGTACCTATAACCAGACCGTTCAGCT
GGATATTACGGCCTTTTTAAAGACCGTAAAGAAAAATAAGCACAAGTTTTATCCG
GCCTTTATTCACATTCTTGCCCGCCTGATGAATGCTCATCCGGAATTTCGTATGGCA
ATGAAAGACGGTGAGCTGGTGATATGGGATAGTGTTCACCCTTGTTACACCGTTTT
CCATGAGCAAACTGAAACGTTTTCATCGCTCTGGAGTGAATACCACGACGATTTC
CGGCAGTTTCTACACATATATTCGCAAGATGTGGCGTGTTACGGTGAAAACCTGGC
CTATTTCCCTAAAGGGTTTATTGAGAATATGTTTTTCGTCTCAGCCAATCCCTGGGT
GAGTTTCACCAGTTTTGATTTAAACGTGGCCAATATGGACAACTTCTTCGCCCCCG
TTTTCACCATGGGCAAATATTATACGCAAGGCGACAAGGTGCTGATGCCGCTGGC
GATTCAGGTTCATCATGCCGTTTGTGATGGCTTCCATGTCGGCAGAATGCTTAATG
AATTACAACAGTACTGCGATGAGTGGCAGGGCGGGGCGTAATTTTTTTAAGGCAG
TTATTGGTGCCCTTAAACGCCTGGGGTAATGACTCTCTAGCTTGAGGCATCAAATA
AAACGAAAGGCTCAGTCGAAAGACTGGGCCTTTCGTTTTATCTGTTGTTTGTCGGT
GAACGCTCTCCTGAGTAGGACAAATCCGCCCTCTAGATTACGTGCAGTCGATGAT
AAGCTGTCAAACATGAGAATTGTGCCTAATGAGTGAGCTAACTTACATTAATTGCG
TTGCGCTCACTGCCCGCTTTCCAGTCGGGAAACCTGTCGTGCCAGCTGCATTAATG
AATCGGCCAACGCGCGGGGAGAGGCGGTTTGCGTATTGGGCGCCAGGGTGGTTTT
TCTTTTCACCAGTGAGACGGGCAACAGCTGATTGCCCTTCACCGCCTGGCCCTGA
GAGAGTTGCAGCAAGCGGTCCACGCTGGTTTGCCCCAGCAGGCGAAAATCCTGT

TTGATGGTGGTTAACGGCGGGATATAACATGAGCTGTCTTCGGTATCGTCGTATCC
CACTACCGAGATATCCGCACCAACGCGCAGCCCGGACTCGGTAATGGCGCGCATT
GCGCCCAGCGCCATCTGATCGTTGGCAACCAGCATCGCAGTGGGAACGATGCCCT
CATTCAGCATTTGCATGGTTTGTTGAAAACCGGACATGGCACTCCAGTCGCCTTCC
CGTTCCGCTATCGGCTGAATTTGATTGCGAGTGAGATATTTATGCCAGCCAGCCAG
ACGCAGACGCGCCGAGACAGAACTTAATGGGCCCGCTAACAGCGCGATTTGCTG
GTGACCCAATGCGACCAGATGCTCCACGCCCAGTCGCGTACCGTCTTCATGGGAG
AAAATAATACTGTTGATGGGTGTCTGGTCAGAGACATCAAGAAATAACGCCGGAA
CATTAGTGCAGGCAGCTTCCACAGCAATGGCATCCTGGTCATCCAGCGGATAGTT
AATGATCAGCCCACTGACGCGTTGCGCGAGAAGATTGTGCACCGCCGCTTTACAG
GCTTCGACGCCGCTTCGTTCTACCATCGACACCACCACGCTGGCACCCAGTTGAT
CGGCGCGAGATTTAATCGCCGCGACAATTTGCGACGGCGCGTGCAGGGCCAGAC
TGGAGGTGGCAACGCCAATCAGCAACGACTGTTTGCCCGCCAGTTGTTGTGCCAC
GCGGTTGGGAATGTAATTCAGCTCCGCCATCGCCGCTTCCACTTTTTCCCGCGTTTT
CGCAGAAACGTGGCTGGCCTGGTTCACCACGCGGGAAACGGTCTGATAAGAGAC
ACCGGCATACTCTGCGACATCGTATAACGTTACTGGTTTCACATTCACCACCCTGA
ATTGACTCTCTTCCGGGCGCTATCATGCCATACCGCGAAAGGTTTTGCACCATTCG
ATGGTGTCGGAATTTCGGGCAGCGTTGGGTCCTGGCCACGGGTGCGCATGATCTA
GAGCTGCCTCGCGCGTTTCGGTGATGACGGTGAAAACCTCTGACACATGCAGCTC
CCGGAGACGGTCACAGCTTGTCTGTAAGCGGATGCCGGGAGCAGACAAGCCCGT
CAGGGCGCGTCAGCGGGTGTTGGCGGGTGTCGGGGCGCAGCCATGACCCAGTCA
CGTAGCGATAGCGGAGTGTATACTGGCTTAACTATGCGGCATCAGAGCAGATTGTA
CTGAGAGTGCACCATATGCGGTGTGAAATACCGCACAGATGCGTAAGGAGAAAA
TACCGCATCAGGCGCTCTTCCGCTTCCTCGCTCACTGACTCGCTGCGCTCGGTCGT
TCGGCTGCGGCGAGCGGTATCAGCTCACTCAAAGGCGGTAATACGGTTATCCACA
GAATCAGGGGATAACGCAGGAAAGAACATGTGAGCAAAAGGCCAGCAAAAGGC
CAGGAACCGTAAAAAGGCCGCGTTGCTGGCGTTTTTCCATAGGCTCCGCCCCCCT
GACGAGCATCACAAAAATCGACGCTCAAGTCAGAGGTGGCGAAACCCGACAGG
ACTATAAAGATACCAGGCGTTTCCCCCTGGAAGCTCCCTCGTGCGCTCTCCTGTTC
CGACCCTGCCGCTTACCGGATACCTGTCCGCCTTTCTCCCTTCGGGAAGCGTGGCG
CTTTCTCATAGCTCACGCTGTAGGTATCTCAGTTCGGTGTAGGTCGTTCGCTCCAA
GCTGGGCTGTGTGCACGAACCCCCCGTTCAGCCCGACCGCTGCGCCTTATCCGGT
AACTATCGTCTTGAGTCCAACCCGGTAAGACACGACTTATCGCCACTGGCAGCAG
CCACTGGTAACAGGATTAGCAGAGCGAGGTATGTAGGCGGTGCTACAGAGTTCTT
GAAGTGGTGGCCTAACTACGGCTACACTAGAAGGACAGTATTTGGTATCTGCGCT
CTGCTGAAGCCAGTTACCTTCGGAAAAAGAGTTGGTAGCTCTTGATCCGGCAAAC
AAACCACCGCTGGTAGCGGTGGTTTTTTTGTTTGCAAGCAGCAGATTACGCGCAG
AAAAAAAGGATCTCAAGAAGATCCTTTGATCTTTTCTACGGGGTCTGACGCTCAG
TGGAACGAAAACTCACGTTAAGGGATTTTGGTCATGAGATTATCAAAAAGGATCT
TCACCTAGATCCTTTTAAATTAAAAATGAAGTTTTAAATCAATCTAAAGTATATATG
AGTAAACTTGGTCTGACAGTTACCAATGCTTAATCAGTGAGGCACCTATCTCAGCG
ATCTGTCTATTTCGTTCATCCATAGTTGCCTGACTCCCCGTCGTGTAGATAACTACG
ATACGGGAGGGCTTACCATCTGGCCCCAGTGCTGCAATGATACCGCGAGACCCAC
GCTCACCGGCTCCAGATTTATCAGCAATAAACCAGCCAGCCGGAAGGGCCGAGC

GCAGAAGTGGTCCTGCAACTTTATCCGCCTCCATCCAGTCTATTAATTGTTGCCGG
GAAGCTAGAGTAAGTAGTTCGCCAGTTAATAGTTTGCGCAACGTTGTTGCCATTGC
TACAGGCATCGTGGTGTCACGCTCGTCGTTTGGTATGGCTTCATTCAGCTCCGGTT
CCCAACGATCAAGGCGAGTTACATGATCCCCCATGTTGTGCAAAAAAGCGGTTAG
CTCCTTCGGTCCTCCGATCGTTGTCAGAAGTAAGTTGGCCGCAGTGTTATCACTCA
TGGTTATGGCAGCACTGCATAATTCTCTTACTGTCATGCCATCCGTAAGATGCTTTT
CTGTGACTGGTGAGTACTCAACCAAGTCATTCTGAGAATAGTGTATGCGGCGACC
GAGTTGCTCTTGCCCGGCGTCAATACGGGATAATACCGCGCCACATAGCAGAACT
TTAAAAGTGCTCATCATTGGAAAACGTTCTTCGGGGCGAAAACTCTCAAGGATCT
TACCGCTGTTGAGATCCAGTTCGATGTAACCCACTCGTGCACCCAACTGATCTTCA
GCATCTTTTACTTTCACCAGCGTTTCTGGGTGAGCAAAAACAGGAAGGCAAAATG
CCGCAAAAAAGGGAATAAGGGCGACACGGAAATGTTGAATACTCATACTCTTCCT
TTTTCAATATTATTGAAGCATTTATCAGGGTTATTGTCTCATGAGCGGATACATATTT
GAATGTATTTAGAAAAATAAACAAATAGGGGTTCCGCGCACATTTCCCCGAAAAG
TGCCACCTGACGTCTAAGAAACCATTATTATCATGACATTAACCTATAAAAATAGG
CGTATCACGAGGCCCTTTCGTCTTCAC

Restriction site
BamH I and Sal I
